# Climate adaptation pathways and the role of social-ecological networks in small-scale fisheries

**DOI:** 10.1038/s41598-022-18668-w

**Published:** 2022-09-15

**Authors:** Diego Salgueiro-Otero, Michele L. Barnes, Elena Ojea

**Affiliations:** 1 Centro de Investigación Mariña, Universidade de Vigo, Future Oceans Lab, Campus Lagoas-Marcosende, 36310 Vigo, Spain; 2 Australian Research Council (ARC) Centre of Excellence for Coral Reef Studies, James Cook University, Townsville, 4811 Australia

**Keywords:** Environmental social sciences, Climate-change adaptation, Sustainability

## Abstract

Climate change is expected to have increasing impacts on marine ecosystems which will threaten the livelihoods and wellbeing of millions of people. Drawing on social-ecological network and sociodemographic data collected via face-to-face interviews with 404 small-scale commercial fishers from 9 Galician communities (Spain), we empirically examine the adaptation pathways that fishers follow when they face hypothetical impacts on their fishery resources and test the role of five social-ecological network structures on fisher’s stated intended responses to such scenarios. Our results show that fishers generally intend to follow a ‘remain—adapt—transform—exit (the fishery)’ pathway when faced with increasing climate impacts. Next, we demonstrate that trust-based bonding ties and ties to informal leaders are associated with a ‘business-as-usual’ strategy. In contrast, communicative bonding ties are associated with adaptive responses, while communicative bridging ties are associated with transformative and exit strategies. Our findings provide key empirical insight that broaden our understanding of the intricate relationship between social networks and adaptive behaviour relevant to social-ecological systems worldwide.

## Introduction

Climate change is expected to have increasing impacts on marine ecosystems worldwide through habitat shifts, new species interactions, and population extirpations^1^. As a consequence, small-scale fisheries (SSF) will experience changes in catch levels, catch composition, and fishing costs that will have cascading impacts on the livelihoods and wellbeing of the millions of people who depend on them ^2,3^. In addition to direct impacts such as disruptions to food supplies and fishing income^4^, climate-induced disruptions to SSF can also result in the exclusion of social groups ^5^, the erosion of social capital ^6^, and the loss of traditions, values, and identity in coastal fishing communities^7–9^. To cope with these impacts, it is critical that coastal fishing communities on the frontlines of climate change have the capacity to adapt ^2,3,5,6,10^.

As global warming accelerates, individuals in coastal fishing communities and elsewhere will need to make decisions regarding whether and how to adapt under different levels of impact. In some cases, making changes to existing practices and behaviours within the current structures of a coupled social-ecological system—often referred to simply as ‘adaptation’^2,11,12^—may be sufficient. Following this definition, in the context of small-scale fisheries an example of adaptation would be changing fishing gear types, targeting new species ^2,13^, or changing markets^14,15^. In more extreme cases, adaptation may not be enough, and people will need to make more fundamental changes that can alter current social-ecological system structures and aid in creating new systems or futures—often referred to as ‘transformation’^2,11,12^. Following this definition, an example of transformation in the context of small-scale fisheries could be engaging in forms of livelihood diversification^12^—though whether and when an action should be considered transformative in nature as opposed to adaptive is still contested and is highly dependent on context^13,16^.

**Figure 1:**
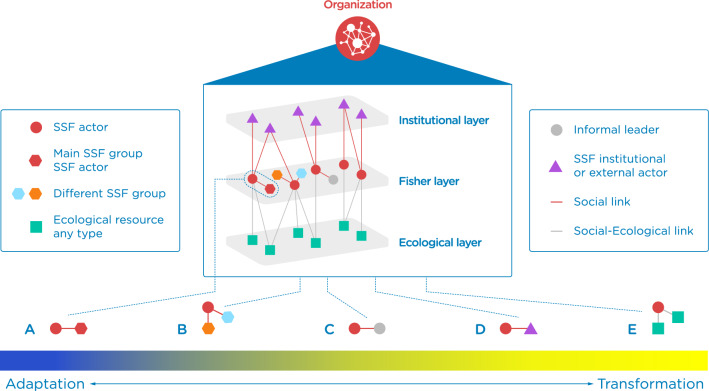
Social and social-ecological network structures hypothesized to support adaptation and transformation. Graphical representation of the social ties that fishers develop with other social actors, such as trust and/or communication (red line), and the social-ecological ties fishers develop with ecological resources (grey line). Ties occur across three layers: (1) institutional layer (with institutional actors represented in triangles); (2) fisher layer (where individuals are represented in circles and fisher groups in hexagons); and (3) ecological layer (with ecological resources represented in squares). These links result in the (**A**–**E**) specific social and social-ecological network structures hypothesized to support adaptation and transformation that we test here, adapted from those in^11^. (**A**) Structure A captures the concept of bonding ties between a fisher (red circle) and the main fishing group the fisher belongs to (red hexagon). (**B**) Structure B depicts bridging ties between a fisher and different fishing groups (yellow and blue hexagons). (**C**) Structure C represents a link between a fisher and informal leaders within their fishing community (grey circle). (**D**) Structure D represents a link between a fisher and institutional actors, such as technical advisors or fishing guards (purple triangles), which are often referred to as linking ties^7^. (**E**) Structure E represents the link between a fisher and their main marine target resources (green squares). On the bottom, the figure illustrates the expected adaptation-transformation continuum based on a recently developed social-structural theory of adaptive behaviour^7^. This gradient shows how organizational structures can predispose individuals to adaptation and/or transformation responses.

Social organization—or the way societies are organized through formal and informal relationships or networks^5^—has long been advocated as a crucial component of adaptive capacity^5,6^. A given organization setting with specific social network structures can facilitate (or constrain) learning, provide access to resources, and reduce the transaction costs associated with coordinating actions^17,18^. Drawing on theories of social organization and risk, recent research has formalized arguments regarding the role of networks on adaptation into a continuum of specific social and social-ecological network structures that are hypothesized to lay the foundation for adaptation and transformation processes^11^. These structures [sometimes referred to as network ‘configurations’^11,19^], are argued to capture specific network capacities that set the stage for effective adaptation and transformation strategies (Fig. 1). For example, developing strong or bonding ties between members of the same group (structure A, Fig. 1) are argued to promote knowledge co-production, trust, reciprocity, and cooperation, all of which can enable fishers to adapt to ecological changes while maintaining the dominant structures and function of the SSF system^17,20,21^. The development of bridging ties between people of different groups (structure B, Fig. 1) can facilitate knowledge sharing, provide access to external resources and support, and may enable innovation—all of which are crucial to adapting to ecological changes and can also enable the development of new perspectives that initiate transformations^11,20–23^. These ties are also sometimes referred to as “weak ties” as they are not as in-depth and enduring (e.g. in terms of frequency of interaction and/or materials that transfer via the tie) as bonding ties^24^. Ties with informal leaders within communities (structure C, Fig. 1) can be critical for activating social capital and facilitating collective action through coordination, both processes that may empower the role of fishers and influence them to consider other potential livelihood alternatives beyond fisheries^20,23–26^. Linking ties (structure D, Fig. 1) between different levels of an organizational structure (e.g., between fishers and policy-makers) help to leverage resources, ideas, and information beyond the level of the community, and can promote legitimacy—thereby enabling real progress toward feasible transformations^11,20,26,27^. Finally, social-ecological network structures (structure E, Fig. 1)—relationships between social actors and their marine target resources—depicts the environmental knowledge and capacity acquired from the connection with different biological marine resources that may enable fishers to detect environmental changes and anticipate future change scenarios^11,12^.

Broadly stated, along this continuum all structures are argued to underpin responses to change, yet bonding network structures (e.g., Fig. 1A) are argued to be more relevant for promoting adaptation; whereas bridging and linking network structures—e.g., connecting different types of actors (e.g., Fig. 1C,D) are argued to be more relevant for facilitating transformations^11^. Though the theoretical foundation for these arguments has been well developed^11^, the framework has yet to be tested empirically. Thus, whether these specific network structures do indeed lay the foundation for adaptation and transformation to occur remains unknown. Similarly, to date, whether and how fishers will adapt, transform, or otherwise respond under different levels of impact or risk associated with climate change has received some attention in the literature, largely from a theoretical standpoint^2,28,29^. However, we lack empirical evidence on individual fisher’s adaptation pathways, such as the series of adaptation responses under different levels of impact.

In this paper, we empirically examine the adaptation pathways that fishers follow when they face incremental negative impacts on the primary resources that support their livelihoods based on their responses to scenarios of climate impacts. Second, we empirically test the role of five organizational social-ecological network structures across the theorized adaptation-transformation continuum (Fig. 1) on fisher’s responses to these climate impact scenarios. Our research questions are thus as follows: (1) what are the response pathways that fishers follow under incremental climate change impact scenarios? and (2) how do organizational social-ecological network structures hypothesized to lay the foundation for adaptation and transformation affect individual’s decisions to adapt, transform, or otherwise respond to these climate impact scenarios?

**Figure 2:**
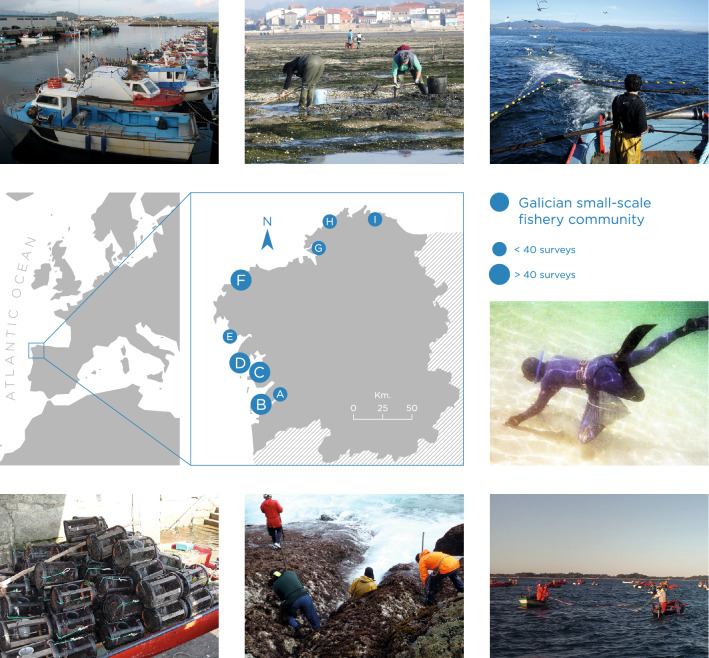
Galician small-scale fisheries communities along the coastline. Case study region and the 9 sampling locations where the research was conducted. Small blue dots show communities where less than 40 surveys were conducted. Big dots identify communities where more than 40 surveys were conducted. Illustration of the diversity of resources, gears, people and socio-economic conditions defines each specific site. From left to right and from top to bottom, pictures show examples of the diversity of Galician SSF: fishing boats at the port, shellfish harvesters on foot, artisanal trawling fishers, divers gathering razor clams, shellfish harvesters on boat, barnacles harvesters and fishing traps on boats.

We empirically investigated these questions across nine SSF communities in Galicia, Northwest Spain (Fig. 2), where climate change is already causing a range of impacts^30,31^. To address our first research question, we developed a structured survey and implemented it via face-to face interviews with 404 fishers in the summer of 2018 (Methods and Supplementary Tables S3–S4). The survey presented five hypothetical climate change scenarios associated with a permanent decrease of the main marine biological resources fishers depended on, impacting catch levels that we linked to an ultimate decrease in individuals’ fishing income by 15%, 30%, 50%, 70%, and 90% in relation to their current income levels. Responses were classified in four categories of strategies: remain (i.e. business-as-usual)^2^, adapt (i.e. changing gears fishing gears and/or target resources but respondent’s continue fishing as an exclusive activity)^12^, transform (i.e. livelihood diversification) and exit the fishery entirely^2^. Capturing fisher’s stated responses to the five increasing impact levels allowed us to depict adaptation pathways.

To answer the second research question, we collected network data on communication, trust, and resource dependency ties (Methods, Table 1 and Supplementary Tables S3–S4) to assess the extent to which respondents are embedded within the structures described in Fig. 1. We also collected data on a range of indicators covering other key aspects (or domains) of adaptive capacity (such as assets, agency, etc.) which may have affected responses that we included as controls in our models^5,6,32^ (see Methods and Supplementary Tables S3–S4). With these data we tested a theoretically-based multinomial multilevel logit mixed-effect model for each hypothetical impact scenario as the dependent variable and the adaptive capacities and network structures as independent variables.

**Table 1:** Dependent and network structure independent variables of the five multinomial multilevel mixed effects models. The full set of variables included in the models (i.e., indicators of adaptive capacity) are further described in Supplementary Tables S1–S3.

Variable name	Measurement
Response to impact scenario (dependent variable)	Adaptation responses to a hypothetical decrease of 15%, 30%, 50%, 70% and 90% of fisher’s income from fishing, following the response decision tree (Supplementary Fig. S1)
Bonding communication (structure A, Fig. 1)	Communication within the SSF group/s of the respondent. Presence (once per year at least) or absence (never)
Bridging communication (structure B, Fig. 1)	Communication with a different SSF group/s (i.e., outside of the respondent’s group/s). Presence (once per year at least) or absence (never)
Linking communication (structure D, Fig. 1)	Communication with institutional actors (i.e., Representative fishers, Technical advisors, Secretary and administrative assistants, Fishing guards, Auction manager, Public regional government). Presence (once per year at least) or absence (never)
Bonding trust (structure A, Fig. 1)	Trust in the SSF group/s the respondent belongs to Presence (any degree of trust) or absence (no degree of trust)
Bridging trust (structure B, Fig. 1)	Trust in SSF groups the respondent does not belong to. Presence (any degree of trust) or absence (no degree of trust)
Linking trust (structure D, Fig. 1)	Trust perceived in SSF institutional actors (Representative fisher, Technical advisor, Secretary and administrative assistants, Fishing guards, Auction manager, Public regional government). Presence (any degree of trust) or absence (no degree of trust)
Communication with informal leaders (structure C, Fig. 1)	Communication with informal SSF leaders who belong to the community Presence (once per year at least) or absence (never). Informal leaders are considered SSF actors that do not take any formal institutional role in the respondent’s community but are recognized as leaders
Dependence on main marine resources (structure E, Fig. 1)	Number of main target resources (in relation to the main target resources in the community)

## Results and discussion

### Adaptation pathways under scenarios of increasing climate impacts

**Figure 3:**
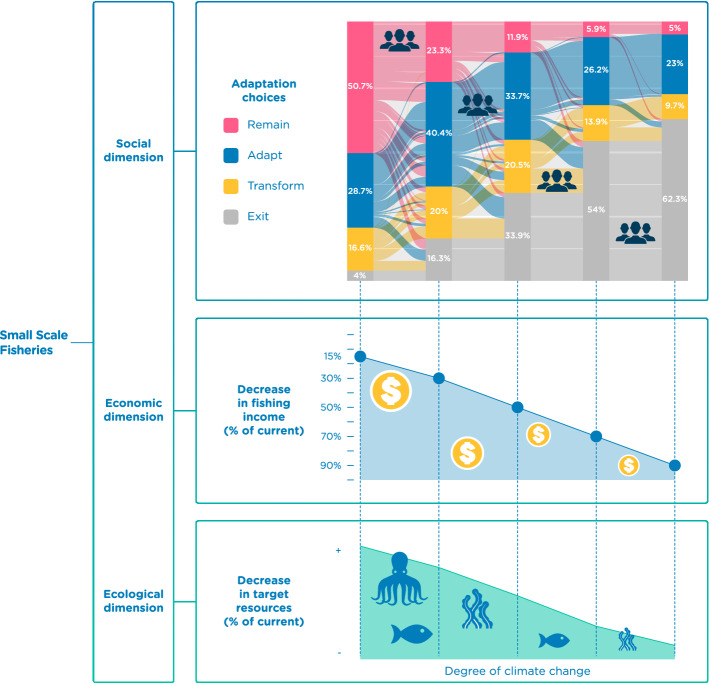
Small-scale fishers’ individual responses to incremental climate change impacts on marine resources (n = 404). Ecological, economic, and social dimensions of the SSF interlinked. The ecological dimension reflects the decrease of target resources. The economic dimension represents a decrease in fishing catches and therefore income, according to climate change impact scenarios presented to respondents. The social dimension above shows our results in terms of the number of respondents who chose each type of response, reflecting the main stated adaptation pathways fisher’s follow when presented with increasing climate impact scenarios.

Our results empirically illustrate the response pathway that fishers follow under incremental change, with an overwhelming majority of fishers following a remain-adapt-transform-exit pathway of some sort, either gradually by moving towards consecutive stages, or abruptly by changing stages in the same direction (Fig. 3). Response pathways occur across five hypothetical scenarios of climate change impacts on the fishing activity. The general trend observed reveals that at lower impact levels, most fishers remain without changes in their livelihoods. As the impact level increases, individuals consecutively adapt and transform, up to an impact level of a 70% decrease in fishing income, where the main observed response is to exit the fishery (Fig. 3).

We found statistically significant differences of individual responses across scenarios (between 15 and 30%, 30% and 50%, and 50% and 70% decrease scenarios *P* < 0.000; between 70 and 90% decrease scenarios *P* < 0.1) (Table S5). Most respondents (50.74%) chose remaining in the fishery under a 15% income decrease scenario, but then moved towards adapt, transform, or exit responses when presented with a 30% decrease scenario. In the 50% decrease scenario, the number of respondents exiting the fishery doubled, though over 50% of respondents still chose to remain in the fishery. In the most extreme scenario of a 90% income decrease, the majority of respondents chose to exit the fishery (62.37%); though some chose to adapt (23.01%), transform (9.65%), and a minority remained (i.e., business-as-usual, 4.95%) (Fig. 3).

Based on our results, a general behavioural pattern emerges when fishers are faced with varying climate-induced impacts to their primary resource base and income source. Specifically, three quarters of the respondents follow a remain-adapt-transform-exit pathway. Approximately 33% follow this pathway gradually, shifting from “remain” to “adapt”, or from “adapt” to “transform” (Fig. 3). In contrast, 43% do not follow the pathway gradually, instead they shift straight from a “remain” to a “transform” response, or from a “remain” to an “exit” response when presented with higher impact levels (Fig. 3). Only a very small minority of fishers (1.5%) did not follow the pathway described above (Fig. 3). Surprisingly, 23% of fishers did not change their response despite the level of impact presented: 11% always chose to adapt, 3% always chose to transform, and 4% always chose to exit (Fig. 3). Importantly, 5% of actors chose to remain in business-as-usual, in some cases even at extreme scenarios of a 90% decrease in income from target resources (Fig. 3).

The persistence of individuals remaining in fisheries even when catches fall beyond economic profitability as demonstrated here is in line with existing research^33–35^, and may be the result of a common characteristic of small-scale fishing as a way of life^36^. This response could also be an example of limited livelihood options outside the fishery and an indicator of potential social-ecological traps, where people remain locked in the SSF activity, falling into economic poverty and reinforcing other detrimental dynamics such as overharvesting^2,5,36,37^. Where impacts on SSF are expected to have such a dramatic impact on fishery income, it will be critical that policy-makers and related professionals work to ensure a sustainable and equitable migration of fishers from SSF towards other suitable livelihoods well before such impacts occur.

**Figure 4:**
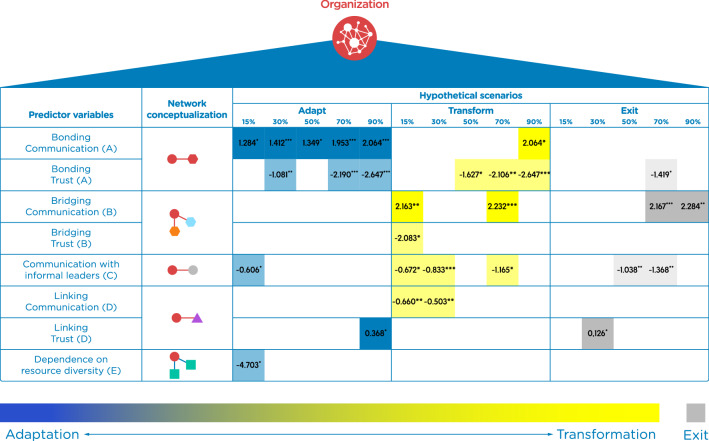
The role of social and social-ecological network structures on responses to incremental climate change impact scenarios. Significant results (*P* < 0.1) from five multinomial multilevel logit mixed-effects models on each climate change hypothetical scenario (− 15%, − 30%, − 50%, − 70%, − 90% in fisheries income). Names and letters of network structures are akin to Fig. 1 and Table 1. Blue, yellow, and grey indicate adaptation, transformation, and exit responses, respectively. Remaining in the fishery—the business-as-usual option—was set as the base category. Brighter colours indicate positive ( +) relationships whereas lighter colours indicate negative (−) relationships. All models controlled for other aspects (i.e., domains) of adaptive capacity; such as assets, agency, learning, flexibility, socio-cognitive factors, and other competing concerns; see Supplementary Table S3 for a description of all covariates included. Full results of the five models are available in Supplementary Tables S7–S11.

We found that organizational social-ecological network structures played a significant role on individual responses to the climate impact scenarios (Fig. 4; see Supplementary Tables S7–S11 for the full model results). Below, we focus on four key findings that were largely consistent across different responses and climate impact scenarios: (1) bonding communication ties and adaptation, (2) trust-based ties and resistance to change, (3) bridging communication ties and transformation, and (4) communication with informal leaders and the status quo. The results of communication and trust-based linking ties, trust-based bridging ties, and resource dependence ties across scenarios were not consistent enough to show a clear pattern and be interpreted in the context of the existing literature. Future research can explore these network structures in greater detail to shed further light on how they may (or may not) be related to responses to climate impacts in different contexts.

### Bonding communication ties and adaptation

Bonding communication ties (Structure A, Fig. 1, Table 1), which capture the communication links between people that belong to the same fishing group (i.e., shellfish gatherers, divers, or purse seine fishers), play a significantly positive role (*P* < 0.1) on ‘adapt’ responses across all impact scenarios (Fig. 4 and Supplementary Tables S7–S11); yet are largely unrelated to other types of responses. Bonding communication ties can explain individual adaptive behaviour in several ways.

First, bonding ties can provide social support as well as a sense of identity and belonging, which may positively influence fishers’ perceptions of efficacy in responding to environmental change^38,39^. Indeed, bonding ties often underpin norms of reciprocity, which can enable people to leverage resources in realizing their response strategies by sharing risk and cost^40,41^, while also providing social support in times of crisis^40,42^. Moreover, those who strongly identify with their group and perceive that the group norm is to engage in adaptation will be more likely to adopt such behaviours^39^. Second, bonding ties can also help people to develop a shared vision (e.g., of the system and the changes it faces), which can promote common agreements and governance practices that enhance adaptation options and actions, potentially safeguarding access to food, income, and ways of living associated with SSFs^43,44^. Finally, bonding ties can facilitate information sharing about adaptation strategies and encourage mutual learning within a group, which may facilitate the adoption of technologies or new strategies that require certain awareness and knowledge^40,42^.

The positive effects of bonding ties in Galician SSFs are observed through several activities that the groups currently perform; such as cleaning the shore and resource surveillance, which are developed by fishers from bonding social groups that target common marine resources; work together; and share practices, time and fishing grounds^45^. Replacing native species by non-native ones to satisfy seafood demand is one of many climate change adaptation examples developed by women in bonding groups that have allowed the Galician shellfish gathers to survive^31^.

Despite the positive role that bonding ties can have on adaptation^46^, it is important to ensure that strong social cohesion stemming from a high level of bonding ties doesn’t disadvantage individuals who are excluded from the group^43^. For instance, bonding social capital developed within groups allows access to certain resources that can support adaptation, such as improved infrastructure, useful tacit knowledge, and support in times of crises^6,43^. Yet groups with high bonding social capital can have a tendency to exclude outsiders, and may shift the brunt of climate impacts to those who do not participate in, or are excluded from the group^43,46^.

Trust-based bonding ties and resistance to change.

Trust-based ties within fishing groups (structure A) suggest a preference for the business-as-usual strategy, especially in the more extreme impact scenarios (Fig. 4 and Supplementary Tables S7-S11). As Galician fishing groups spend time together for fishing, training, decision making, and vision sharing, many fishers may develop strong trust-based relationships with each other. According to the risk literature, trust in this context may serve to reduce the complexity of a situation—especially when people lack knowledge about a hazard, their risk judgments tend to be based on the degree to which they trust responsible risk managers^47^. In other words, when trust is high, individual risk perception may be low, which can impede risk preparedness and vice versa^43,47,48^. This effect could explain why trust-based bonding ties are negatively related to adaptive responses in our study, i.e., trust-based ties may cause people to perceive adaptive strategies as unnecessary. In addition, as trust relationships enhance cohesion and activate future transactional and functional ties between individuals already connected^49^, a high level of trustful bonding ties could also pose a barrier to initiating a transformation due to potentially constraining social norms or mental lock-ins^11^. For instance, a high level of bonding social capital could be a negative force if it creates a “we” versus “them” in-group/out-group mentality^50^.

Based on these results, trust-based bonding ties are related to remaining in a business-as-usual strategy, in contrast to the role of bonding communication ties on adaptive responses. Although much research has focused on the role of bonding social capital on adaptation^6^ and the beneficial role of bonding ties for the collective management of resources and community development more broadly^44^, existing research warns that in some cases bonding links can reduce resilience^26^. Despite these contrasts, there has been little research that distinguishes between different types of bonding relationships. Our research has demonstrated that this distinction is potentially very important, since communication-based bonding ties are positively related to adaptation, yet trust-based bonding ties are negatively related to both adaptation and transformation (particularly under higher levels of climate-induced impacts). This result is a crucial distinction to take into account in the design and planning of adaptation measures, so that the potential consequences of these two different social processes can be considered.

### Bridging communication ties and transformation

The third key result is that bridging communication ties (structure B) were positively related to transform and exit strategies (*P* > 0.10), particularly in higher impact scenarios. In Galicia, a complex community-based management system has been developing through centuries based on the rich marine environment. As some small-scale fishers target several species by different gears in different habitats under inter-related official rules, they develop bridging ties with different fishing groups^23^. Our findings demonstrate that under climate impact conditions, those small-scale fishers that communicate with different fishing groups are more likely to diversify their livelihoods, or even exit fisheries all together.

Our results are aligned with the existing literature that explains that bridging ties allow people to access external resources, manage critical information, exchange diverse knowledge, and enhance innovation by bridging in new perspectives to deal with (social-ecological) changes^11,20,22,23,26,27^. Thus, fishers holding bridging ties may combine information about different practices, resources, and environmental conditions; and analyze alternative governance arrangements – all facilitating more transformative responses to environmental change. In the Cambodian agriculture context for example, individuals who adopt transformative practices were found to link diverse sources of knowledge that helped them tackle uncertainty and change^26^. Research on Australian agriculture systems similarly demonstrates that weak social ties and access to broader sources of knowledge empower individuals to plan and implement novel transformative strategies and options in the face of climate change^51^.

In relation to recent research, our results uncover a potential conflict for high climate impact scenarios where bridging ties may be needed to facilitate transformations (and exit), yet where bonding ties promoting remaining or adapting within SSFs prevail. Specifically, work on how communities and governance systems deal with risk has suggested that low-risk situations tend to be associated with the emergence of bridging structures in communication networks, whereas high-risk situations tend to be associated with the emergence of bonding structures (the ‘risk hypothesis’)^17^. Yet our results suggest that bridging ties are critical for encouraging transformative responses to climate impacts—particularly under high impact (and thus high-risk) scenarios. It may therefore be necessary to encourage the establishment (and maintenance) of bridging ties (structure B) within SSF systems even as climate risks intensify in order to avoid undesirable social-ecological traps. As a form of social capital, a balance of different types of social ties have generally been shown to contribute to the development of robust adaptive capacity and enhance collective action at the community level^6,50^.

### Communication with informal leaders and the status quo

Our results demonstrate that communication-based ties with informal leaders tend to have a negative relationship with all types of responses across various impact scenarios (Fig. 4), suggesting that communicative ties with informal leaders are associated with a preference to maintain the status-quo. These intriguing results stand in contrast to existing research that emphasizes the transformative role that leaders can play in SSF communities when faced with climate or other social and ecological change (Fig. 1)^25,52^. There are several potential explanations for this result. The first is that fishers who communicate with informal leaders may not feel the need to make changes to their behavior because they believe that leaders bear responsibility^43^ and trust them to manage risks^26^. Some of the social processes described above in reference to bonding trust-based ties may be also be relevant here, since risk judgments tend to be based on the degree to which people trust responsible risk managers. Though we did not directly measure trust in informal leaders in this study, research on social networks demonstrates that social interaction (such as communication) across authority gradients tends to embody norms of respect which can result in trusting relationships^53^. Thus, relationships with informal leaders may reduce fisher’s risk perception despite the impacts they are facing^43,47^, causing them to choose a ‘business-as-usual’ strategy. Fishers who trust leaders to manage risks may also feel their individual adaptation redundant in relation to leaders’ practices^43^.

A second potential explanation is that fishers who develop communicative ties with informal leaders may feel more invested in, and feel they have more control or power over SSF dynamics, causing them to become reluctant to change their behaviour; so they remain in the business-as-usual strategy despite increasing climate impacts^43^. Being more invested in a fishery can make it more difficult to change fishing practices and/or engage in other livelihood options. Existing research also indicates that those in powerful positions in social-ecological systems can become resistant to change, since they likely stand to lose from such changes, which often involve shifts in power^12,22,54^. At worst, influential individuals can contribute to elite capture—defined as situations where certain individuals dominate decision making and in turn disproportionally improve their access to benefits from common-pool fishery resources. In such instances, ‘leadership’ can work against the greater benefit of the community^55^. We did not explicitly measure perceptions of power or elite capture in our research, and power dynamics are generally under-emphasized and poorly understood in global change science more broadly^56^. It will be critical that future research gains a better understanding of the distribution of costs and benefits with respect to winners and losers from adaptation decision-making in order to avoid situations of elite capture and steer system transformations when necessary.

Finally, in some communities, informal leaders (often referred to as ‘opinion leaders’) may be popular and influential because they are conservative in their beliefs and their behavior adheres to local social norms and values^26^. In other words, being recognized by peers as an informal leader does not imply progressivity^26^. Indeed, recent studies have found that local ‘opinion leaders’ often do not act as role models for the adoption of new or progressive practices because they chose not to adopt the practice themselves^26,57^. Thus, in our case small-scale fishers who often communicate with informal leaders may be more likely to remain taking a business-as-usual approach when faced with various climate change impact scenarios if they assume that informal leaders would also be resistant to changing their behaviour. In this regard, power dynamics may play a key role in individual (and community) adaptation, and further research should be conducted on how leadership and power dynamics interact to shape adaptation in SSF^57^.

### The role of context and other factors shaping responses to climate change

The insights provided by this research need to be understood as only one piece of the adaptation puzzle. Adaptation is a context and place-dependent process which can be affected by many of factors^58–63^. Indeed, in addition to the organizational social-ecological network structures which formed the primary focus of our inquiry, we found that adaptation was also influenced by covariates capturing heterogeneity among fishers and important aspects of socioeconomic context (such as income, fishing assets, and livelihood diversity) as well as agency (household size and participation in decision making) (see Table S3–S4). Adaptation to climate impacts in small-scale fishing communities are often nested across scales, and can also be influenced by existing policy and historical contexts which were outside of the scope of our study (i.e. existing regulations, property rights, and institutional arrangements). These factors have been shown to be important in SSF communities^58,59,64^ and future research that seeks to determine how these factors interact with organizational social-ecological network structures and other factors typically included in conceptualizations of adaptive capacity^5^ to influence adaptation pathways is needed.

## Conclusion

In this paper we have conducted an empirical analysis of small-scale fishers’ individual adaptation responses to a set of hypothetical climate change impact scenarios. Applying a network approach, we tested the effect of social-ecological network structures on fishers’ individual responses across a continuum of increasing climate impact scenarios.

Our first primary contribution is novel empirical evidence demonstrating that individual fishers follow a common response pathway that ranges from remaining in business-as-usual, to adapting, transforming, and exiting the fishery. Second, we contribute to adaptation theory by providing the first empirical test of a theoretical framework developed to disentangle the relationship between specific social/social-ecological network structures and individual adaptive responses across a continuum of change^11^.

Our results largely confirm initial hypotheses^11^ that link bonding network structures with adaptation and bridging network structures with transformation strategies. Specifically, we found that bonding communication ties—facilitators of mutual learning and providers of social support—are positively related to adaptive responses; while bridging communicative ties—which can provide access to novel ideas and resources—are positively related to transformative and exit responses. However, diverging from existing theoretical arguments^11^ we found that trust-based bonding ties were related to a business-as-usual approach, thus shedding critical light on the differential effects that communication-based and trust-based ties may have on individual responses to change. Our results also provide novel empirical evidence that communicative ties with informal leaders are associated with maintaining the status quo, rather than acting as a catalyst for adaptation and/or transformation. This evidence confirms that social-ecological network structures (Fig. 1) form the necessary—but not sufficient—conditions for adaptation and transformation to occur^11^.

Our novel analysis demonstrates that the social relationships people develop with others are a key contributor to adaptive human capacities, and are significantly related to the type of adaptive responses chosen by individuals facing climate change impacts. Our research focused on SSFs in Galicia, Spain, and therefore our results may be context dependent. However, many SSFs around the globe are similarly structured in that they are multi-species, multi-gear artisanal fisheries comprised of several different overlapping groups of fishers that are heavily involved in fisheries management^65,66^ and are similarly impacted by climate change^67,68^. We therefore believe the insights provided by our results have implications for SSFs more broadly, as well as for cross sectorial adaptation theory. Existing research based on empirical data of fishery landings, revenues, and governance show that maintaining spatial diversification, diversity in economic opportunities, and flexibility in the fishing sector facilitates adaptation and the sustainability of fishing communities facing environmental changes and uncertain futures^64,69–71^. We conclude that an advanced understanding of the role of social-ecological network structures on adaptive responses can further help to improve strategies for navigating change in fishing communities and other social-ecological systems.

## Materials and methods

### Site selection

We conducted this research in Galicia (NW Spain), where small-scale fisheries (SSF) are socially, historically, and economically important^72,73^. The SSF system is roughly composed by 13,500 fishers organized into 63 fisher guilds (*Confrarías*)^72^*.* Guilds are the main geographical and administrative entity for SSF, placed on a material infrastructure in the fishing community and overseeing management (see Supporting Information for more details). Therefore, each guild is linked to a single community and every community is linked to a single guild. The Galician SSF is highly diverse in terms of target resources (73 taxa), fishing gears (43 different exploitation techniques) and regulations^72,73^. The tight cultural attachment of Galician fishing communities to the marine environment, the complex social context of fisher guilds as the core of SSF communities^72,73^, and the emerging evidence of climate change impacts on marine habitats^30,31^ makes the Galician SSF a suitable case study for this research.

### Summary of empirical strategy

Fishing communities were selected in a stratified manner in order to represent different sizes of fisher guilds, different main target resources and fisher groups, different geographical locations (two criteria: shelter side and open side of coastline covering the south, middle, and north ranges of the coastline), presence/lack of protected areas in their territories, and additional demographic variables such as gender and age. We designed a structured survey and pre-tested it with focus groups in two separate SSF communities in Galicia, with 13 and 12 participants each. The final refined survey was implemented via face-to face interviews in other nine representative fishing communities along the coastline. Five trained interviewers performed face-to-face interviews during the month of July 2018 by randomly approaching fishers at ports and fish markets of the guilds; resulting in a total sample of 404 fishers, representing 12–70% of total fishers in each community (Supplementary Table S1). The average length of the surveys was 45 min. Fishers responded to a set of climate impact scenarios that would change their income from fishing. We also collected important context-dependent information on social relationships (bonding, bridging, and linking ties; ties to informal leaders), dependence on resources, other adaptive capacity indicators and competing concerns (Supplementary Tables S1–S4), which we describe below.

### Responses to climate change

The survey instrument presented five hypothetical impact scenarios as described in the main text, which were selected from existing work^35^ and further refined in the focus groups. We directly linked the decrease in marine biological resources (an ecological impact) with an equal proportional decrease in catch and income in our scenarios (an economic impact) in order to standardize the impact that fishers would face (i.e., we sought to avoid individuals making varying predictions about fluctuating prices due to a potential increase in demand for scarcer resources, which information from our focus groups suggested that some fishers would have expected prices to rise with increasing resource scarcity). We also ask fishers to disregard institutional factors (such as licenses and access permits) in the hypothetical impact scenario in order to remove any potential bias in our sample that may have been due to institutional arrangements (Fig. S1.). To classify the individual responses to each of the hypothetical scenarios, we used a decision tree where we depicted 7 different options (shown in Supplementary Fig. S1). Based on expert knowledge and our focus group results, interviewers were guided through this decision tree in a two-step process. First, fishers were asked if they would remain within the fishery they were already involved in (“SSF as a unique professional activity”, Supplementary Fig. S1), diversify their livelihood by engaging in a different activity in addition to fishing (“Combine SSF with complementary activity”, Supplementary Fig. S1), or exit the fishery (“Exit the fishery”, Supplementary Fig. S1). If they responded that they would exit, we marked this as “new position” (option 7, Supplementary Fig. S1), and stopped there. However, if they responded that they would remain in the fishery, we then asked if they would keep the same resources and gears (option 1, Supplementary Fig. S1); specialize on fewer resources or target new resources or use new gears (option 2, Supplementary Fig. S1), or change their target resources and gears completely (option 3, Supplementary Fig. S1). If they responded that they would combine their fishing activities with a complementary activity, we followed up asking if they would keep the same resources and gears (option 4, Supplementary Fig. S1); specialize on fewer resources, target new resources, or use new gears (option 5, Supplementary Fig. S1), or change their target resources and gears completely (option 6, Supplementary Fig. S1). In line with existing literature^2,12^, the seven options in the decision tree were grouped in a final set of four choices: remain (option 1), adapt (options 2–3), transform (options 4–6), and exit (option 7). We statistically compared the patterns followed by respondents under each climate change impact scenario applying the chi-square test (Supplementary Table S5) and plotted the adaptation pathways (Fig. 1) using the “dplyr”^74^ (1.0.2 version), “tidyr”^75^ (1.0.0 version), “reshape2”^76^ (1.4.3 version),“ggplot2”^77^ (3.3.2 version) and “ggalluvial”^78^ (0.11.1 version) packages in R version 3.6.1 (R Development Core Team 2019).

### Constructing social and social-ecological network structures

We collected information regarding social and social-ecological ties hypothesized to underpin adaptive behaviour^11^ using our surveys. Bonding groups of fishers are typically created around SSF activities that have common regulations, fishing gears (*métiers)*^79^, fishing practices, and the cultural context. Based on these factors and supervised by the focus groups, we identified 10 different small-scale fishing groups for the Galician SSF: shellfish gatherers on foot, shellfish gatherers on boat, demersal/specific resources on foot, demersal/specific resources on boat, divers, trap fishers, gillnet fishers, bait fishers, small-scale purse seine fishers and artisanal trawling fishers (Fig. 2). Following this definition of bonding groups, bridging ties are common in the Galician SSF; i.e., due to the diversity and complexity of this system, some fishers belong to several fishing groups because they target several species from different ecosystems using different gears (*métiers*) and permits, thus providing them an opportunity to act as bridges (commonly known as brokers) in the SSF system.

Within the Galician SSF context, we identify formal (institutional recognized) and informal leadership (fishers without institutional recognition). We identified six main common profiles for institutional actors for the nine sampled communities (e.g., community representative, technical stocks assessments and management plans assistant, secretary, guard watching, auction manager, government representative). Ties to institutional actors were identified as linking ties. Depending on the context of the communities, these profiles can overlap in the same person. Beyond these positions, the integration of a diversity of profiles, perspectives, and interests in the Galician SSF imply the presence of multiple informal leaders, with aligned and opposite voices that may arise in the same SSF community. We identified and quantified ties to informal leaders, as identified by respondents.

To capture the social structures described in Fig. 1, we used data from our surveys regarding communication-based, trust-based, and fishing-based ties (Supplementary Table S3). For measuring bonding, bridging, and linking relationships (structures A, B, and D; Table 1), fishers were asked to respond to communication-based and trust-based questions such as “For the following official positions (institutional actors) and fishing groups (10 groups above mentioned), could you tell us the frequency of communication-based relationships you have with the them?” and “For the following official positions (institutional actors) and fishing groups (10 groups above mentioned), could you tell us the degree of trust you have with them?”, respectively. For the case of communicative relationships with informal leaders (structure C; Table 1), we first asked respondents to think of three people that they felt had influence in the fishery. We then asked fishers the frequency in which they communicated with these individuals (if at all). In order to prevent overlap with our linking ties measure (structure D, Table 1), only ties with informal leaders were included for the structure C. For our measure of social-ecological ties (structure E, Table 1), we asked fishers to identify the name and number of main marine resources they target.

For communication-based and trust-based ties, we measured the number of existing ties (i.e., presence/absence) from respondents to other groups/actors. For bonding ties (structure A), we calculated the proportion of groups (in relation of the existing groups within the community) that each respondent belonged to and identified communication-based and trust-based ties with. With respect to bridging ties (structure B), we calculated the proportional number of groups (in relation to the existing groups within the community) that an actor does not belong to, and identified communication-based and trust-based ties with. To capture communication-based ties with informal leaders (structure C), we first looked at the leaders that fishers identify in the survey, and select those that do not hold a formal position in the system as informal leaders. Then we created a binary variable accounting for the ties with these informal leaders. To quantify linking ties (structure D), we accounted for the existing institutional profiles of each community (maximum of six) to then, calculate the number of these institutional actors with whom the fisher keeps communication-based and trust-based ties. And finally, we also quantified the dependence of SSF actors on marine resources (structure E) by counting the number of the main marine resources that each respondent targets and converting this number into a proportion of the total main resources in the community, as reported by respondents. Since the hypothetical scenarios assume an equal level of impact in every main resource a fisher has, structure E does not account for the fishers’ capacity to rely on less impacted resources they fish in comparison with the more-impacted resources^11^. Despite this, structure E still captures the environmental knowledge acquired by their ties with biological species, and the capacity to work with several resources (fishing-grounds, gears, licenses and knowledge) that fishers have, all those key aspects for facing climate change and promote transformation^11^.

### Accounting for other aspects of adaptive capacity

A large body of recent research on adaptation from across the social sciences emphasizes six domains of adaptive capacity in addition to other competing concerns which are likely to impact how people respond to climate change^5,6,80^. Based on this research we developed 24 key social, economic, and social-ecological indicators to capture the six broad domains of adaptive capacity [defined as: (1) the assets people own or have access to, (2) the flexibility to switch between strategies, (3) the learning capacity to recognize change and assess response strategies, (4) the way society is organized with formal and informal relationships through social networks and institutions (the primary focus of this paper, and described above), (5) the subjective socio-cognitive dimensions that include the risk attitudes, and (6) the agency to mobilize all domains to actively respond to climate stressors^5,6^], and competing concerns (i.e. perception of inequality and gender)^6^. These are further described in Supplementary Tables S3–S4.

### Modelling procedure

To disentangle the links between social-ecological network structures and responses to climate change impact scenarios, we fit five multinomial, multilevel logit mixed-effect models—one per climate change impact scenario (Fig. 1), with the 24 predictors included as numerical, ordinal and binary factors described in Supplementary Tables S3–S4 (adaptive capacity factors, competing concerns, and social-ecological interactions conceptualized as network structures), and one outcome variable with four categories (climate change responses taken by fishers: remain, adapt, transform, and exit). For the models, 29 of 404 total observations had to be dropped due to missing values, resulting in a sample size of 375 fishers. The fishing community was included in our models as a random effect to account for potential differences across sites. Variance inflation factors indicated no issues with multicollinearity among our predictors (Supplementary Table S6). The model was run with robust variance estimates to account for any potential issues of non-independence related to the manner in which our social and social-ecological network structures were conceptualized and calculated. All models were analysed in Stata/ IC 16. The model estimates the significance of the variables using a maximum marginal likelihood. In general terms, the contribution of fixed components is higher than the contribution of random components to the variability of the dependent variable.

### Caveats

Limitations of this research are implicit to the reality of the case study context; the use of fishers’ perceptions and hypothetical scenarios; the assumption of equal proportional decrease between abundance of resources, catches, and profit; the chosen adaptive capacity framework predictors used in the multinomial models^5,80^; and the specific description and calculation of predictors (Supplementary Tables S3–S4). To analyse the social-ecological network structures, we classified people within generalized working groups based on their main resources and asked people about their communication and trust-based ties based on these groups, when the existing theoretical social-structural framework for adaptation largely discussed ties between individual actors^11^, and when people might also belong to other working groups regarding secondary target resources that we did not quantify. Finally, our analysis focused on the quantity of social interactions. We leave for future work further empirical investigations that focus on the quality of communication and trust-based ties in underpinning adaptive behaviour.

### Ethics statement

Participation was voluntary and responses anonymized. Informed consent was obtained from all subjects and/or their legal guardian(s). The experimental protocol was approved by the University of Vigo. Also, a formal agreement with the federal Galician SSF association allow reaching the communities and the acceptance of the fisher guilds to be part of the research project. The whole process has followed the ethical guidelines and requirements set by H2020.

## Supplementary Information

Supplementary Information.

## Data Availability

The datasets generated during and/or analysed during the current study are not publicly available due to privacy/ethical restrictions but they are available from the corresponding author on reasonable request.
