# Supplementary material for: Climate adaptation pathways and the role of social-ecological networks in small-scale fisheries
Source: Sci Rep. 2022 Sep 15;12:15526. doi: 10.1038/s41598-022-18668-w (PMC9478087; doi:10.1038/s41598-022-18668-w)
Supplement: Supplementary file 1 — Supplementary Information. [file 41598_2022_18668_MOESM1_ESM.pdf]

Supplementary Information for
Climate adaptation pathways and the role of social-ecological networks in small-scale fisheries

Diego Salgueiro-Otero, Michele L. Barnes, Elena Ojea

Material and Methods

Site selection
In the Galician SSF context, each community is strongly attached to a fisher guild, the main geographical and administrative entity based on a top-down governance system. Within fisher guilds, fishers carry out their fishing activity under several types of management regulations, including territorial use rights for fisheries (TURFs), total allowable catches (TACs), individual transferable quotas (ITQs), fishing effort-based, gears-based, area-based limitations (e.g. open shellfish-gathering areas or gillnet-based fisheries targeting hake and mackerel) and minimum landing size restrictions[1,2,3,4]. Broadly speaking, SSF communities have the option to manage some coastal areas individually by themselves or commonly with several SSF communities under exploitation and management plans (*plans xerais de explotación* and *plans de xestión*) accepted by the official Galician government. Fishing areas can be exclusive historic territory of communities (*autorizacións*) or open territories to practice fishing activities under official legislation (*zonas de libre marisqueo*). For the latter, the Galician government establishes general guidelines defining resource-specific limits of catches for the unregulated territories by any community management plan. In these cases, only fishers whose fishing licenses under the Galician rules allow them to exploit the resources living in that areas can access to those biological natural resources.

Table S1. Number of fishers and surveys conducted in each Galician SSF community (from south to north).

SSF community	Fishers	Nº of surveys	Coverage of communities with surveys (%)
Community A	247	38	15.38
Community B	261	65	24.90
Community C	542	78	14.39
Community D	349	60	17.19
Community E	40	28	70.00
Community F	111	42	37.84
Community G	140	30	21.43
Community H	258	33	12.79
Community I	55	30	54.55
Total	2003	404	-

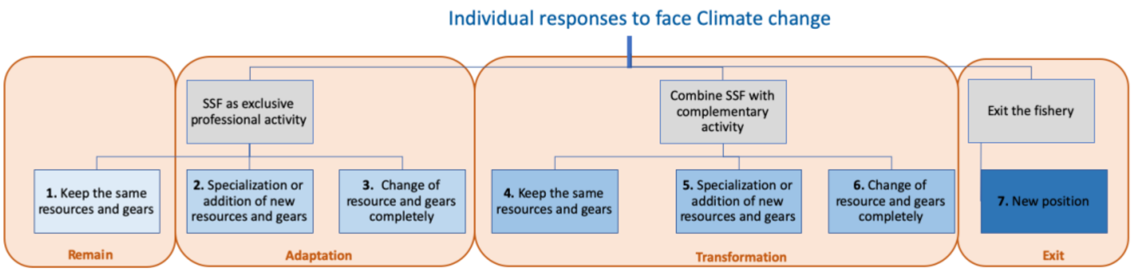

Fig. S1. Decision tree guiding individual fisher responses to climate change impacts.

After the question "In this section we will show a set of hypothetical scenarios under which your main resources (mentioned before) will decrease in the sea permanently due to environmental conditions. So, from now on, imagine a decrease of your catches due to these impacts. This change in catches happens in the main marine species you fish/gather, but the rest of resources don´t have to suffer the same trend necessarily. If we ignore the restrictions of licenses and access permits, what would you do to face each impact?", the decision tree was presented to fishers in order to guide them to response facing climate change events.

Table S2. Summary of dependent variables (n = 404).

Climate impact scenario	Category of responses	Nº of responses (%)
- 15% scenario	Nothing	205 (59.77%)
	Adaptation	116 (33.82%)
	Transformation	67 (1.75%)
	Exit	16 (4.66%)
- 30% scenario	Nothing	94 (23.27%)
	Adaptation	163 (40.35%)
	Transformation	81 (20.05%)
	Exit	66 (16.34%)
- 50% scenario	Nothing	48 (11.88%)
	Adaptation	136 (33.66%)
	Transformation	83 (20.54%)
	Exit	137 (33.91%)
- 70% scenario	Nothing	24 (5.94%)
	Adaptation	106 (26.24%)
	Transformation	56 (13.86%)
	Exit	218 (53.96%)
- 90% scenario	Nothing	20 (4.95%)
	Adaptation	93 (23.02%)
	Transformation	39 (9.65%)
	Exit	252 (62.38%)

Table S3. Description of independent variables arranged by adaptive capacity domains.

Domain	Variable name	Description	Question in survey	References
Assets	Fishing assets	Number of fishing assets	Indicate which of the following assets you have*: car, boat, boat (with engine), own gears, bank account, internet access.*	5,9,11
	Income	Range of total income	Which is the income range that better fits with your situation in relation to the whole set of work activities you are currently involved? *0-500€, 500-1000€, 1000-1500€, 1500-2000€, 2000-2500€, 2500-3000€, 3000-3500€, >3500€ monthly*	6,10,13
Flexibility	Livelihood diversity	Number of complementary income activities	Nº of current complementary labor activity	5,9,11
	Fisher identity	Degree of pride in being a fisher	Indicate the level of agreement with the following sentence (Likert-scale): *I feel proud of being a fisher and/or shellfish gatherer in this place*	5,6
	Job attachment	Degree of job attachment	Indicate the level of agreement with the following sentence (Likert-scale): *I will not change to other job although I would have other better job option*	5,10,15
	Age	Age of the respondent	How old are you?	8,9,10
Learning	SES knowledge	Degree of knowledge about the SSF system as a social-ecological system	Indicate the level of agreement with the following sentence (Likert-scale): *I know very well the rules and law that affect my fishing, the institutional organisms which regulate my fishing activity, the species behavior/dynamics in the environment, and the life style of fisher-folk*	5,6
	Training activities	Degree of participation in activities in the fishing community. Average of answers from the 3 types of organized activities for each respondent.	For the organized activities in the Confraría during the last year, indicate your degree of participation (1- *I am a very active participant, 2- I participate a little, 3- I do not participate*):<br>• Participation in training activities (courses, etc.)<br>• Participation in auction and markets activities (transport, classification, quality, control, etc.)<br>Participation in activities to improve the state of environment and resource management (harvest, removals, hatcheries, cleaning activities, etc.)	5,6
	Fishing experience	Months spent on the SSF activity	During your life, how much time did you dedicate to artisanal/SSF?	6,12,14

Agency	Household size	Nº of members in the household	How many members are in your household?	5,9,15
	Participation decision making	Degree of participation in decision-making in the fishing guild	Indicate the level of agreement with the following sentence (Likert-scale): *I can take part in the decision-making process that manages my resources in the Confraría*	6,8,9
Socio-Cognitive	Risk attitude	Degree of risk taken in the labor activity	Indicate the level of agreement with the following sentence (Likert-scale): *Sometimes I cross the line in my job and put myself in danger*	5,8,15
Competing concerns	Inequality	Degree of perceived inequality between fisher groups	Indicate the level of agreement with the following sentence (Likert-scale): *In my Confraría, I consider my sector to be in an inequality situation in relation to other sectors in the same Confraría*	6,15
	Gender	Binary indicator (men, women) of the respondent as a proxy of biological data based on the physical appearance	Gender by the physical appearance of respondents. Women were coded as 0 and men were coded as 1.	9,15
Social Organization	Social capital	Degree of perceived social capital at work, in terms of workers taking care of each other	Indicate the level of agreement with the following sentence (Likert-scale): *At work, everyone is taking care of everyone*	5,8,10
	Bonding communication (structure A)	Communication with the SSF group/s of the respondent. Presence (once per year at least) or absence (never).	For the following official positions and fishing groups within SSF activity, could you tell us the frequency in which you communicate with them? [a]	5,7,8
	Bridging communication (structure B)	Communication with a different SSF group/s (i.e., outside of the respondent's group/s). Presence (once per year at least) or absence (never).	For the following official positions and fishing groups within SSF activity, could you tell us the frequency in which you communicate with them? [a]	5,7,8
	Linking communication (structure D)	Communication with institutional actors (i.e., Representative fishers, Technical advisors, Secretary and administrative assistants, Fishing guards, Auction manager, Public regional government). Presence (once per year at least) or absence (never).	For the following official positions (institutional actors) and fishing groups within SSF activity, could you tell us the frequency in which you communicate with them? [a]	5,7,8
	Bonding trust (structure A)	Trust in SSF groups the respondent belongs to. Presence (any degree of trust) or absence (no degree of trust).	For the following official positions and fishing groups within SSF activity, could you tell us the degree of trust you have with them? [a]	5,7,8
	Bridging trust (structure B)	Trust in SSF groups the respondent does not belongs to. Presence (any degree of trust) or absence (no degree of trust).	For the following official positions and fishing groups within SSF activity, could you tell us the degree of trust you have with them? [a]	5,7,8
	Linking trust (structure D)	Trust perceived in SSF institutional actors (Representative fisher, Technical advisor, Secretary and administrative assistants, Fishing guards, Auction	For the following official positions (institutional actors) and fishing groups within SSF activity, could you tell us the degree of trust you have with them? [a]	5,7,8

		manager, Public regional government). Presence (any degree of trust) or absence (no degree of trust).		
	Communication with informal leaders (structure C)	Communication with informal SSF leaders who belong to the community. Presence (once per year at least) or absence (never). In this work informal leaders are considered SSF actors that do not take any formal institutional role in the respondent's community	1-Now, think of three people with more influence in SSF activity. Mention the fishing groups or official positions (institutional actors) they belong to. 2-For these three people, could you tell us the frequency in which you communicate with them?	5,7,8
	Dependence on main marine resources (structure E)	Number of main target resources (in relation with the main target resources in his/her community)	Currently, which are your main target resources?	5,7,8
-	Community	SSF community (municipality) where the respondent is involved in SSF activities (random effect variable)	Place/Name of Confraría	12,14

[a] Official positions (institutional actors) were: SSF actor representative, scientific-technical advisor, secretary and administrative staff, fishing guard, auction manager, and public regional government. Social groups were: shellfish gatherers on foot, shellfish gatherers on boat, benthic gatherers on foot, benthic gatherers on boat, benthic gatherers divers, fishing trap, fishing gillnet, fishing lines, fishing trawl, fishing seine.

Table S4. Summary statistics of independent variables.

Domain	Variable name	Type of response	Category of response	Mean	Standard deviation	Range	Nº responses (%)	NA
Assets	Fishing assets	Discrete	-	4.359	1.176	1-6	-	0
	Income	Ordinal	0-500€	-	-	-	21 (5.43%)	17
			500-1000€	-	-	-	121 (31.27%)	
			1000-1500€	-	-	-	147 (37.98%)	
			1500-2000€	-	-	-	62 (16.02%)	
			2000-2500€	-	-	-	15 (3.88%)	
			2500-3000€	-	-	-	12 (3.10%)	
			3000-3500€	-	-	-	5 (1.29%)	
			>3500€ per month	-	-	-	4 (1.03%)	
Flexibility	Livelihood diversity	Discrete	-	0.191	0.441	0-2	-	0
	Fisher identity	5-Likert	Very much agree	-	-	-	8 (1.99%)	2
			Disagree	-	-	-	9 (2.24%)	
			Neither disagree nor agree	-	-	-	17 (4.23%)	
			Agree	-	-	-	136 (3.83%)	
			Very much agree	-	-	-	232 (57.71%)	
	Job attachment	5-Likert	Very much agree	-	-	-	122 (30.85%)	2
			Disagree	-	-	-	137 (34.08%)	
			Neither disagree nor agree	-	-	-	31 (7.71%)	
			Agree	-	-	-	73 (18.16%)	
			Very much agree	-	-	-	39 (9.70%)	
	Age	Discrete	-	43.437	10.010	19-70	-	1
Learning	SES knowledge	5-Likert	Very much agree	-	-	-	12 (2.99%)	3
			Disagree	-	-	-	24 (5.99%)	
			Neither disagree nor agree	-	-	-	84 (20.95%)	
			Agree	-	-	-	193 (48.13%)	
			Very much agree	-	-	-	88 (21.95%)	
	Training activities	Continuous indicator (from average of 3 ordinal variables)	-	2.006	0.584	0.67-3	-	2
	Fishing experience	Continuous	-	234.683	152.737	0-804	-	0
Agency	Household size	Discrete	-	3.322	1.288	1-10	-	0
	Participation decision making	5-Likert	Very much agree	-	-	-	48 (11.97%)	3
			Disagree	-	-	-	63 (15.71%)	

			Neither disagree nor agree	-	-	-	38 (9.48%)	
			Agree	-	-	-	178 (44.39%)	
			Very much agree	-	-	-	74 (18.45%)	
Socio-Cognitive	Risk attitude	5-Likert	Very much agree	-	-	-	76 (18.86%)	1
			Disagree	-	-	-	119 (29.53%)	
			Neither disagree nor agree	-	-	-	45 (11.17%)	
			Agree	-	-	-	93 (23.08%)	
			Very much agree	-	-	-	70 (17.37%)	
Competing concerns	Inequality	5-Likert	Very much agree	-	-	-	57 (14.21%)	3
			Disagree	-	-	-	118 (29.43%)	
			Neither disagree nor agree	-	-	-	47 (11.72%)	
			Agree	-	-	-	107 (26.68%)	
			Very much agree	-	-	-	72 (17.96%)	
	Gender	Binary	Female	-	-	-	78 (19.31%)	0
			Male				326 (80.69%)	
Social Organization	Social capital	5-Likert	Very much agree	-	-	-	33 (8.21%)	2
			Disagree	-	-	-	92 (22.89%)	
			Neither disagree nor agree	-	-	-	43 (10.70%)	
			Agree	-	-	-	148 (36.82%)	
			Very much agree	-	-	-	86 (21.39%)	
	Bonding communication (structure A)	Continuous	-	0.7036139	0.4144467	0-1	-	0
	Bridging communication (structure B)	Continuous	-	0.3906188	0.2825344	0-1	-	0
	Linking communication (structure D)	Continuous	-	4.712871	1.254958	0-6	-	0
	Bonding trust (structure A)	Continuous	-	0.6859158	0.4276689	0-1	-	0
	Bridging trust (structure B)	Continuous	-	0.3942327	0.3048201	0-1	-	0
	Linking trust (structure D)	Continuous	-	4.371287	1.649353	0-6	-	0
	Communication with informal leaders (structure C)	Binary	Yes	-	-	-	165 (40.84%)	0
			No	-	-	-	239 (59.16%)	
	Dependence on main marine resources (structure E)	Continuous	-	0.1218564	0.08454971	0-1	-	0

Random effect	Community	Random effect	-	-	-	-	-	0

Table S5. Comparison of responses to different climate change impact scenarios.

Comparing scenarios	X-squared	Degrees of freedom	p-value
-15% and -30%	80.937	3	< 0.000
-30% and -50%	41.858	3	< 0.000
-50% and -70%	35.831	3	< 0.000
-70% and -90%	6.7146	3	0.081

Table S6. Variance Inflation Factors.

Variable	VIF	1/VIF
Bridging trust (Configuration B)	3.85	0.260
Bonding trust (Configuration A)	3.80	0.263
Bridging communication (Configuration B)	3.63	0.276
Bonding communication (Configuration A)	3.55	0.281
Linking communication (Configuration D)	2.40	0.417
Linking trust (Configuration D)	2.32	0.431
Fishing experience	2.18	0.458
Age	2.05	0.489
Gender	1.58	0.634
Income	1.47	0.681
Community	1.41	0.708
Fishing assets	1.38	0.725
Training activities	1.30	0.767
Fishing identity	1.30	0.770
Participation decision making	1.28	0.782
Job attachment	1.25	0.799
Inequality	1.25	0.803
Risk attitude	1.23	0.815
SES knowledge	1.22	0.821
Dependence on marine resource diversity (Configuration E)	1.18	0.847
Social capital	1.14	0.877
Communication with informal leaders (Configuration C)	1.12	0.892
Livelihood diversity	1.11	0.899
Household size	1.11	0.901
Mean VIF	1.84	

Multinomial multilevel logit mixed effects models

Table S7. Full multinomial model results: 15% decrease scenario**.**

Variable predictor	Coefficient (β)	Robust standard error	Z-value	P>|z|	95% Confidence interval	
**Choice: Nothing**						
Base outcome						
**Choice: Adaptation**						
Bonding communication	1.284	0.536	2.40	0.017	0.234	2.334
Bridging communication	-0.339	0.699	-0.48	0.628	-1.709	1.031
Linking communication	0.002	0.135	0.02	0.986	-0.263	0.268
Communication informal leaders	-0.606	0.346	-1.75	0.080	-1.284	0.072
Bonding trust	-0.793	0.549	-1.44	0.149	-1.868	0.283
Bridging trust	0.392	1.072	0.37	0.715	-1.709	2.493
Linking trust	-0.038	0.087	-0.43	0.666	-0.208	0.133
Dependence on marine resources	-4.703	2.520	-1.87	0.062	-9.642	0.236
Gender	0.367	0.615	0.60	0.551	-0.838	1.571
Fisher identity	0.004	0.303	0.01	0.990	-0.590	0.598
Social capital	-0.298	0.128	-2.33	0.020	-0.549	-0.048
Inequality	0.033	0.088	0.38	0.706	-0.140	0.206
Risk attitude	0.020	0.143	0.14	0.889	-0.261	0.301
Participation in decision making	-0.072	0.152	-0.48	0.635	-0.371	0.226
Job attachment	-0.086	0.104	-0.82	0.411	-0.290	0.119
SES knowledge	-0.326	0.085	-3.82	0.000	-0.493	-0.159
Income	-0.188	0.151	-1.24	0.214	-0.484	0.109
Household size	0.005	0.074	0.07	0.947	-0.140	0.149
Fishing assets	0.130	0.104	1.25	0.211	-0.074	0.334
Age	-0.046	0.013	-3.45	0.001	-0.072	-0.020
Livelihood diversity	-0.347	0.562	-0.62	0.537	-1.449	0.755
Training activities	0.191	0.332	0.57	0.565	-0.459	0.840
Fishing experience	0.027	0.019	1.39	0.164	-0.011	0.065
M1[confraria]	1.000	constrained	.			
_cons	3.173	2.146	1.48	0.139	-1.034	7.379
**Choice: Transformation**						
Bonding communication	0.095	0.633	0.15	0.880	-1.146	1.336
Bridging communication	2.163	0.887	2.44	0.015	0.423	3.902
Linking communication	-0.660	0.343	-1.93	0.054	-1.332	0.012
Communication informal leaders	-0.672	0.370	-1.82	0.069	-1.398	0.053
Bonding trust	-0.596	0.756	-0.79	0.431	-2.078	0.887
Bridging trust	-2.083	1.096	-1.90	0.057	-4.231	0.065
Linking trust	0.379	0.240	1.58	0.114	-0.092	0.849
Dependence on marine resources	1.039	2.581	0.40	0.687	-4.020	6.098
Gender	-0.751	0.515	-1.46	0.144	-1.760	0.257
Fisher identity	0.513	0.219	2.35	0.019	0.084	0.941
Social capital	-0.308	0.257	-1.20	0.230	-0.812	0.195
Inequality	-0.072	0.132	-0.54	0.587	-0.330	0.187
Risk attitude	0.081	0.155	0.52	0.604	-0.224	0.385
Participation in decision making	-0.237	0.197	-1.20	0.230	-0.623	0.149
Job attachment	-0.056	0.148	-0.38	0.705	-0.347	0.235
SES knowledge	0.006	0.165	0.03	0.973	-0.317	0.328
Income	-0.117	0.141	-0.83	0.406	-0.392	0.159
Household size	-0.096	0.158	-0.61	0.545	-0.407	0.215
Fishing assets	0.164	0.142	1.15	0.248	-0.115	0.443
Age	-0.0563	0.027	-2.12	0.034	-0.108	-0.004
Livelihood diversity	2.859	0.552	5.18	0.000	1.777	3.942
Training activities	-0.163	0.406	-0.40	0.688	-0.958	0.633
Fishing experience	0.009	0.018	0.47	0.638	-0.027	0.045
M1[confraria]	1.000	constrained	.			
_cons	2.367	2.672	0.89	0.376	-2.871	7.604
**Choice: Exit**						
Bonding communication	0.730	1.139	0.64	0.521	-1.502	2.962
Bridging communication	0.717	1.816	0.40	0.693	-2.843	4.277
Linking communication	0.098	0.347	0.28	0.777	-0.581	0.777
Communication informal leaders	-0.862	0.721	-1.19	0.232	-2.275	0.552
Bonding trust	-0.026	1.294	-0.02	0.984	-2.563	2.510
Bridging trust	-2.895	1.897	-1.53	0.127	-6.614	0.824
Linking trust	-0.204	0.274	-0.75	0.456	-0.741	0.332

Dependence on marine resources	3.020	1.964	1.54	0.124	-0.830	6.870
Gender	19.59	0.877	22.35	0.000	17.870	21.306
Fisher identity	0.461	0.371	1.24	0.215	-0.267	1.189
Social capital	-0.139	0.253	-0.55	0.582	-0.635	0.356
Inequality	0.260	0.176	1.47	0.141	-0.086	0.605
Risk attitude	0.003	0.241	0.01	0.991	-0.469	0.475
Participation in decision making	0.097	0.432	0.22	0.823	-0.750	0.943
Job attachment	-0.392	0.108	-3.64	0.000	-0.604	-0.181
SES knowledge	-0.440	0.234	-1.88	0.060	-0.898	0.019
Income	-0.308	0.538	-0.57	0.567	-1.362	0.746
Household size	0.245	0.179	1.37	0.170	-0.105	0.596
Fishing assets	0.006	0.132	0.05	0.962	-0.252	0.265
Age	-0.004	0.044	-0.10	0.919	-0.091	0.082
Livelihood diversity	-0.375	0.861	-0.44	0.663	-2.062	1.312
Training activities	0.264	0.668	0.40	0.692	-1.046	1.574
Fishing experience	0.014	0.037	0.38	0.708	-0.059	0.087
M1[confraria]	1.000	constrained	.			
_cons	-21.86	0.184	-6.86	0.000		
var (M1[confraria])	0.186	0.171	1.09		0.031	1.130

Measures of fit

Log likelihood	-336.660
Log pseudolikelihood	-331.876

Table S8. Full multinomial model results: 30% decrease scenario.

Variable predictor	Coefficient (β)	Robust standard error	Z-value	P>|z|	95% Confidence interval	
**Choice: Nothing**						
Base outcome						
**Choice: Adaptation**						
Bonding communication	1.412	0.492	2.87	0.004	0.446	2.377
Bridging communication	-1.169	0.896	-1.30	0.192	-2.924	0.587
Linking communication	0.012	0.118	0.10	0.921	-0.220	0.243
Communication informal leaders	-0.118	0.334	-0.35	0.725	-0.773	0.538
Bonding trust	-1.081	0.458	-2.36	0.018	-1.978	-0.184
Bridging trust	0.284	0.711	0.40	0.689	-1.109	1.678
Linking trust	-0.117	0.184	-0.63	0.526	-0.477	0.244
Dependence on marine resources	-2.388	3.072	-0.78	0.437	-8.409	3.633
Gender	0.730	0.439	1.66	0.096	-0.130	1.589
Fisher identity	-0.017	0.409	-0.04	0.967	-0.819	0.785
Social capital	-0.149	0.160	-0.93	0.352	-0.462	0.165
Inequality	0.075	0.139	0.54	0.591	-0.197	0.346
Risk attitude	0.049	0.123	0.40	0.688	-0.192	0.290
Participation in decision making	0.157	0.182	0.86	0.390	-0.201	0.514
Job attachment	0.035	0.113	0.31	0.756	-0.186	0.256
SES knowledge	-0.399	0.177	-2.26	0.024	-0.745	-0.053
Income	-0.045	0.163	-0.27	0.785	-0.365	0.276
Household size	0.122	0.099	1.23	0.217	-0.072	0.316
Fishing assets	0.133	0.123	1.08	0.280	-0.108	0.374
Age	-0.004	0.025	-0.17	0.862	-0.053	0.044
Livelihood diversity	-0.196	0.437	-0.45	0.654	-1.051	0.660
Training activities	0.477	0.463	1.03	0.303	-0.430	1.384
Fishing experience	-0.002	0.021	-0.11	0.914	-0.044	0.040
M1[confraria]	1.000	(constrained)	.			
_cons	0.355	2.451	0.14	0.885	-4.449	5.159
**Choice: Transformation**						
Bonding communication	0.182	0.604	0.30	0.763	-1.002	1.366
Bridging communication	0.039	1.267	0.03	0.975	-2.445	2.523
Linking communication	-0.503	0.248	-2.03	0.043	-0.990	-0.016
Communication informal leaders	-0.833	0.300	-2.78	0.005	-1.421	-0.245
Bonding trust	-0.955	0.940	-1.02	0.310	-2.798	0.887
Bridging trust	-0.450	1.291	-0.35	0.728	-2.980	2.081
Linking trust	0.213	0.287	0.74	0.457	-0.349	0.775
Dependence on marine resources	1.609	2.941	0.55	0.584	-4.156	7.374
Gender	-0.660	0.625	-1.06	0.291	-1.886	0.566
Fisher identity	0.364	0.385	0.94	0.345	-0.392	1.119
Social capital	-0.355	0.233	-1.52	0.127	-0.811	0.101
Inequality	0.015	0.195	0.08	0.938	-0.366	0.397
Risk attitude	0.178	0.086	2.06	0.039	0.009	0.347
Participation in decision making	-0.103	0.254	-0.41	0.685	-0.601	0.395
Job attachment	-0.057	0.207	-0.28	0.782	-0.462	0.348
SES knowledge	-0.038	0.366	-0.10	0.918	-0.754	0.679
Income	-0.030	0.137	-0.22	0.824	-0.298	0.237
Household size	0.179	0.158	1.13	0.257	-0.130	0.488
Fishing assets	-0.058	0.137	-0.42	0.672	-0.326	0.210
Age	-0.023	0.024	-0.95	0.343	-0.070	0.024
Livelihood diversity	2.327	0.516	4.51	0.000	1.315	3.338
Training activities	0.314	0.401	0.78	0.433	-0.471	1.100
Fishing experience	-0.020	0.021	-0.98	0.328	-0.060	0.020
M1[confraria]	1.000	(constrained)	.			
_cons	1.939	2.457	0.79	0.430	-2.877	6.754
**Choice: Exit**						
Bonding communication	-0.349	0.916	-0.38	0.704	-2.145	1.447
Bridging communication	0.065	0.916	0.07	0.943	-1.730	1.860
Linking communication	-0.100	0.143	-0.69	0.488	-0.381	0.182
Communication informal leaders	-0.644	0.417	-1.54	0.123	-1.461	0.174
Bonding trust	-0.331	1.001	-0.33	0.741	-2.292	1.630
Bridging trust	-1.511	1.251	-1.21	0.227	-3.963	0.940
Linking trust	0.126	0.073	1.74	0.083	-0.016	0.269
Dependence on marine resources	0.899	3.789	0.24	0.812	-6.527	8.326

Gender	0.235	0.706	0.33	0.739	-1.148	1.619
Fisher identity	0.034	0.416	0.08	0.936	-0.781	0.849
Social capital	-0.082	0.199	-0.41	0.680	-0.472	0.308
Inequality	0.129	0.148	0.87	0.385	-0.162	0.419
Risk attitude	-0.111	0.106	-1.04	0.297	-0.318	0.097
Participation in decision making	-0.064	0.208	-0.31	0.759	-0.471	0.343
Job attachment	-0.423	0.165	-2.57	0.010	-0.745	-0.100
SES knowledge	-0.342	0.201	-1.70	0.089	-0.737	0.052
Income	-0.125	0.159	-0.79	0.429	-0.436	0.185
Household size	0.121	0.155	0.78	0.437	-0.184	0.425
Fishing assets	0.073	0.207	0.35	0.724	-0.332	0.478
Age	-0.071	0.035	-2.01	0.044	-0.140	-0.002
Livelihood diversity	0.626	0.431	1.45	0.146	-0.219	1.471
Training activities	-0.084	0.503	-0.17	0.867	-1.071	0.902
Fishing experience	0.045	0.020	2.22	0.027	0.005	0.085
M1[confraria]	1.000	(constrained)	.			
_cons	5.439	2.841	1.91	0.055	-0.128	11.007
var (M1[confraria])	0.255	0.386	0.66		0.013	4.927

Measures of fit

Log likelihood	-409.012
Log pseudolikelihood	-405.844

Table S9. Full multinomial model results: 50% decrease scenario.

Variable predictor	Coefficient (β)	Robust standard error	Z-value	P>|z|	95% Confidence interval	
**Choice: Nothing**						
Base outcome						
**Choice: Adaptation**						
Bonding communication	1.349	0.728	1.85	0.064	-0.077	2.775
Bridging communication	-1.051	1.244	-0.84	0.398	-3.490	1.387
Linking communication	-0.046	0.222	-0.21	0.837	-0.481	0.390
Communication informal leaders	-0.136	0.484	-0.28	0.778	-1.084	0.812
Bonding trust	-1.388	0.848	-1.64	0.102	-3.050	2.688
Bridging trust	0.602	1.064	0.57	0.571	1.483	2.688
Linking trust	0.012	0.227	0.05	0.958	-0.433	0.457
Dependence on marine resources	-3.702	2.549	-1.45	0.146	-8.699	1.294
Gender	0.678	0.242	2.80	0.005	0.204	1.151
Fisher identity	-0.098	0.391	-0.25	0.802	-0.864	0.667
Social capital	-0.009	0.189	-0.05	0.961	-0.864	0.360
Inequality	-0.0115	0.106	-0.11	0.914	-0.220	0.197
Risk attitude	0.113	0.069	1.63	0.103	-0.023	0.248
Participation in decision making	-0.010	0.176	-0.06	0.954	-0.354	0.334
Job attachment	-0.222	0.160	-1.39	0.164	-0.535	0.091
SES knowledge	0.015	0.209	0.07	0.944	-0.395	0.424
Income	-0.029	0.174	-0.17	0.867	-0.370	0.312
Household size	0.147	0.101	1.46	0.145	-0.051	0.346
Fishing assets	0.216	0.212	1.02	0.309	-0.200	0.632
Age	0.002	0.032	0.07	0.947	-0.060	0.064
Livelihood diversity	-0.288	0.659	-0.44	0.663	-1.580	1.005
Training activities	0.317	0.466	0.68	0.496	-0.596	1.230
Fishing experience	0.005	0.024	0.20	0.842	-0.042	0.052
M1[confraria]	1.000	(constrained)	.			
_cons	0.272	2.789	0.10	0.922	-5.194	5.738
**Choice: Transformation**						
Bonding communication	1.258	0.962	1.31	0.191	-0.627	3.144
Bridging communication	0.478	1.061	0.45	0.652	-1.602	2.558
Linking communication	-0.298	0.222	-1.34	0.180	-0.734	0.138
Communication informal leaders	-0.508	0.415	-1.22	0.222	-1.322	0.307
Bonding trust	-1.627	0.988	-1.65	0.099	-3.563	0.309
Bridging trust	0.037	1.278	0.03	0.977	-2.468	2.542
Linking trust	0.227	0.252	0.90	0.367	-0.267	0.722
Dependence on marine resources	2.212	2.329	0.95	0.342	-2.352	6.777
Gender	-0.780	0.520	-1.50	0.133	-1.798	0.239
Fisher identity	0.143	0.395	0.36	0.718	-0.632	0.918
Social capital	0.011	0.211	0.05	0.960	-0.404	0.425
Inequality	-0.023	0.158	-0.14	0.887	-0.333	0.288
Risk attitude	0.402	0.095	4.23	0.000	0.216	0.588
Participation in decision making	0.028	0.148	0.19	0.853	-0.263	0.318
Job attachment	-0.225	0.310	-0.73	0.468	-0.832	0.383
SES knowledge	0.635	0.231	2.75	0.006	0.183	1.087
Income	-0.047	0.197	-0.24	0.813	-0.432	0.339
Household size	0.221	0.203	1.09	0.276	-0.176	0.617
Fishing assets	0.233	0.213	1.10	0.273	-0.184	0.651
Age	0.003	0.023	0.14	0.892	-0.041	0.047
Livelihood diversity	1.643	0.533	3.08	0.002	0.598	2.687
Training activities	0.273	0.293	0.93	0.350	-0.300	0.847
Fishing experience	-0.037	0.027	-1.40	0.162	-0.089	0.015
M1[confraria]	1.000	(constrained)	.			
_cons	-4.009	1.484	-2.70	0.007	-6.918	-1.101
**Choice: Exit**						
Bonding communication	-0.702	0.975	-0.72	0.471	-2.614	1.209
Bridging communication	1.102	1.593	0.69	0.489	-2.020	4.225
Linking communication	-0.166	0.249	-0.67	0.504	-0.655	0.322
Communication informal leaders	-1.038	0.474	-2.19	0.028	-1.966	-0.109
Bonding trust	-0.120	1.247	-0.10	0.923	-2.564	2.324
Bridging trust	-1.937	1.760	-1.10	0.271	-5.387	1.513
Linking trust	0.153	0.201	0.76	0.445	-0.240	0.547
Dependence on marine resources	1.135	3.015	0.38	0.707	-4.774	7.044

Gender	1.162	0.730	1.59	0.111	-0.268	2.592
Fisher identity	0.033	0.473	0.07	0.944	-0.894	0.961
Social capital	-0.045	0.237	-0.19	0.851	-0.510	0.420
Inequality	0.047	0.139	0.34	0.735	-0.225	0.319
Risk attitude	0.003	0.089	0.04	0.970	-0.171	0.177
Participation in decision making	-0.169	0.226	-0.75	0.454	-0.611	0.273
Job attachment	-0.529	0.217	-2.44	0.015	-0.953	-0.104
SES knowledge	0.019	0.254	0.08	0.939	-0.479	0.517
Income	-0.147	0.145	-1.02	0.310	-0.432	0.137
Household size	0.215	0.161	1.34	0.180	-0.100	0.530
Fishing assets	0.217	0.239	0.91	0.365	-0.252	0.686
Age	-0.042	0.025	-1.66	0.096	-0.090	0.007
Livelihood diversity	0.720	0.612	1.18	0.240	-0.480	1.920
Training activities	0.147	0.514	0.28	0.776	-0.861	1.154
Fishing experience	0.024	0.016	1.53	0.127	-0.007	0.054
M1[confraria]	1.000	(constrained)	.			
_cons	3.341	3.231	1.03	0.301	-2.991	9.674
var (M1[confraria])	2.11E-32	0.000	0.11		0.000	0.000

Measures of fit

Log likelihood	-405.567
Log pseudolikelihood	-401.041

Table S10. Full multinomial model results: 70% decrease scenario.

Variable predictor	Coefficient (β)	Robust standard error	Z-value	P > |z|	95% Confidence interval	
**Choice: Nothing**						
Base outcome						
**Choice: Adaptation**						
Bonding communication	1.953	0.541	3.61	0.000	0.893	3.014
Bridging communication	0.993	0.875	1.13	0.257	-0.722	2.708
Linking communication	-0.324	0.334	-0.97	0.332	-0.979	0.331
Communication informal leaders	-0.736	0.659	-1.12	0.264	-2.026	0.555
Bonding trust	-2.190	0.777	-2.82	0.005	-3.714	-0.667
Bridging trust	-1.015	0.886	-1.15	0.252	-2.753	0.722
Linking trust	0.287	0.189	1.52	0.129	-0.084	0.659
Dependence on marine resources	-2.122	5.317	-0.40	0.690	-12.544	8.300
Gender	1.355	0.578	2.34	0.019	0.221	2.488
Fisher identity	-0.604	0.731	-0.83	0.409	-2.038	0.830
Social capital	0.403	0.344	1.17	0.242	-0.272	1.078
Inequality	-0.085	0.238	-0.36	0.722	-0.550	0.381
Risk attitude	-0.101	0.135	-0.74	0.457	-0.366	0.165
Participation in decision making	-0.079	0.293	-0.27	0.789	-0.653	0.496
Job attachment	-0.797	0.390	-2.04	0.041	-1.563	-0.032
SES knowledge	0.231	0.319	0.72	0.470	-0.395	0.857
Income	0.071	0.288	0.25	0.804	-0.493	0.636
Household size	0.257	0.249	1.03	0.303	-0.232	0.745
Fishing assets	0.398	0.337	1.18	0.237	-0.262	1.057
Age	-0.008	0.068	-0.13	0.901	-0.141	0.124
Livelihood diversity	-0.895	0.747	-1.20	0.231	-2.358	0.568
Training activities	1.199	0.800	1.50	0.134	-0.370	2.767
Fishing experience	-0.022	0.052	-0.42	0.676	-0.125	0.081
M1[confraria]	1.000	(constrained)	.			
_cons	2.716	6.320	0.43	0.667	-9.671	15.104
**Choice: Transformation**						
Bonding communication	1.214	0.740	1.64	0.101	-0.237	2.665
Bridging communication	2.232	0.624	3.58	0.000	1.008	3.455
Linking communication	-0.354	0.441	-0.80	0.422	1.219	0.511
Communication informal leaders	-1.165	0.666	-1.75	0.080	-2.471	0.141
Bonding trust	-2.106	1.028	-2.05	0.041	-4.121	-0.091
Bridging trust	-1.741	1.272	-1.37	0.171	-4.234	0.752
Linking trust	0.246	0.271	0.91	0.364	-0.285	0.776
Dependence on marine resources	3.463	5.946	0.58	0.560	-8.191	15.116
Gender	-0.090	0.716	-0.13	0.900	-1.493	1.313
Fisher identity	-0.839	0.733	-1.14	0.253	-2.276	0.599
Social capital	0.491	0.314	1.56	0.118	-0.125	1.107
Inequality	-0.386	0.213	-1.81	0.070	-0.804	0.031
Risk attitude	-0.054	0.143	-0.38	0.706	-0.334	0.226
Participation in decision making	-0.202	0.199	-1.02	0.310	-0.593	0.188
Job attachment	-0.633	0.367	-1.72	0.085	-1.353	0.087
SES knowledge	0.715	0.249	2.87	0.004	0.227	1.203
Income	0.115	0.319	0.36	0.718	-0.510	0.740
Household size	0.625	0.322	1.94	0.052	-0.005	1.255
Fishing assets	0.583	0.393	1.48	0.138	-0.187	1.352
Age	0.025	0.058	0.43	0.668	-0.089	0.139
Livelihood diversity	0.995	0.646	1.54	0.123	-0.271	2.261
Training activities	1.590	0.602	2.64	0.008	0.409	2.771
Fishing experience	-0.116	0.057	-2.04	0.041	-0.228	-0.004
M1[confraria]	1	(constrained)	.			
_cons	-0.770	4.389	-0.18	0.861	-9.371	7.832
**Choice: Exit**						
Bonding communication	0.607	0.688	0.88	0.378	-0.741	1.955
Bridging communication	2.167	0.787	2.75	0.006	0.624	3.709
Linking communication	-0.279	0.336	-0.83	0.406	-0.937	0.379
Communication informal leaders	-1.368	0.617	-2.22	0.027	-2.577	-0.159
Bonding trust	-1.419	0.854	-1.66	0.096	-3.093	0.254
Bridging trust	-1.712	1.159	-1.48	0.139	-3.983	0.558
Linking trust	0.215	0.212	1.02	0.310	-0.200	0.630
Dependence on marine resources	2.691	5.854	0.46	0.646	-8.782	14.164

Gender	1.431	0.749	1.91	0.056	-0.036	2.898
Fisher identity	-0.515	0.751	-0.69	0.492	-1.987	0.956
Social capital	0.331	0.358	0.93	0.355	-0.370	1.032
Inequality	-0.161	0.217	-0.74	0.457	-0.586	0.264
Risk attitude	-0.056	0.133	-0.42	0.671	-0.317	0.204
Participation in decision making	-0.194	0.263	-0.74	0.461	-0.710	0.322
Job attachment	-0.910	0.385	-2.36	0.018	-1.664	-0.156
SES knowledge	0.285	0.255	1.12	0.263	-0.214	0.784
Income	-0.039	0.293	-0.13	0.895	-0.613	0.536
Household size	0.340	0.279	1.22	0.223	-0.207	0.887
Fishing assets	0.324	0.332	0.98	0.329	-0.326	0.974
Age	-0.049	0.048	-1.01	0.310	-0.143	0.045
Livelihood diversity	0.425	0.556	0.76	0.445	-0.666	1.515
Training activities	1.107	0.649	1.71	0.088	-0.165	2.378
Fishing experience	-0.003	0.037	-0.08	0.939	0.075	0.070
M1[confraria]	1.000	(constrained)	.			
_cons	5.718	5.519	1.04	0.300	-5.099	16.535
var (M1[confraria])	0.804	0.843	0.95		0.103	6.285

Measures of fit

Log likelihood	-331.123
Log pseudolikelihood	-329.831

Table S11. Full multinomial model results: 90% decrease scenario.

Variable predictor	Coefficient (β)	Robust standard error	Z-value	P > |z|	95% Confidence interval	
**Choice: Nothing**						
Base outcome						
**Choice: Adaptation**						
Bonding communication	2.064	0.630	3.27	0.001	0.829	3.299
Bridging communication	1.244	1.379	0.90	0.367	-1.459	3.948
Linking communication	-0.224	0.275	-0.82	0.415	-0.763	0.314
Communication informal leaders	-0.364	0.503	-0.72	0.469	-1.350	0.622
Bonding trust	-2.647	0.795	-3.33	0.001	-4.204	-1.089
Bridging trust	-0.015	0.834	-0.02	0.986	-1.649	1.620
Linking trust	0.368	0.216	1.71	0.088	-0.055	0.790
Dependence on marine resources	-4.007	5.490	-0.73	0.466	-14.767	6.754
Gender	1.863	0.654	2.85	0.004	0.581	3.144
Fisher identity	-0.982	0.879	-1.12	0.264	-2.705	0.741
Social capital	0.387	0.332	1.17	0.244	-0.264	1.039
Inequality	-0.103	0.140	-0.73	0.464	-0.377	0.172
Risk attitude	-0.084	0.129	-0.65	0.514	-0.338	0.169
Participation in decision making	-0.052	0.494	-0.10	0.917	-1.020	0.917
Job attachment	-0.670	0.327	-2.05	0.041	-1.311	-0.029
SES knowledge	0.191	0.387	0.49	0.621	-0.567	0.949
Income	0.381	0.280	1.36	0.173	-0.167	0.929
Household size	0.312	0.375	0.83	0.406	-0.423	1.047
Fishing assets	0.304	0.399	0.76	0.445	-0.477	1.086
Age	0.050	0.032	1.58	0.114	-0.012	0.112
Livelihood diversity	-0.932	0.742	-1.26	0.209	-2.387	0.522
Training activities	1.350	0.755	1.79	0.074	-0.129	2.829
Fishing experience	-0.071	0.032	-2.18	0.029	-0.134	-0.007
M1[confraria]	1.000	(constrained)	.			
_cons	0.485	3.572259	0.14	0.892	-6.516	7.487
**Choice: Transformation**						
Bonding communication	2.124	1.257	1.69	0.091	-0.338	4.587
Bridging communication	1.686	1.127	1.50	0.135	-0.524	3.895
Linking communication	0.069	0.356	0.19	0.846	-0.629	0.767
Communication informal leaders	-0.760	0.651	-1.17	0.243	-2.036	0.516
Bonding trust	-3.271	1.323	-2.47	0.013	-5.863	-0.679
Bridging trust	0.442	1.071	0.41	0.680	-1.657	2.540
Linking trust	0.168	0.236	0.71	0.477	-0.295	0.631
Dependence on marine resources	1.158	5.797	0.20	0.842	-10.204	12.521
Gender	0.226	0.802	0.28	0.778	-1.347	1.799
Fisher identity	-1.118	0.849	-1.32	0.188	-2.782	0.546
Social capital	0.534	0.265	2.01	0.044	0.014	1.055
Inequality	-0.551	0.123	-4.47	0.000	-0.793	-0.309
Risk attitude	0.239	0.141	1.69	0.090	-0.038	0.516
Participation in decision making	-0.129	0.356	-0.36	0.716	-0.827	0.568
Job attachment	-0.406	0.344	-1.18	0.239	-1.081	0.270
SES knowledge	0.638	0.481	1.33	0.185	-0.305	1.580
Income	0.430	0.339	1.27	0.205	-0.234	1.094
Household size	0.499	0.461	1.08	0.279	-0.405	1.403
Fishing assets	0.522	0.365	1.43	0.153	-0.195	1.238
Age	0.071	0.043	1.63	0.103	-0.014	0.155
Livelihood diversity	0.679	0.544	1.25	0.212	-0.388	1.746
Training activities	1.475	0.710	2.08	0.038	0.084	2.867
Fishing experience	-0.166	0.048	-3.46	0.001	-0.260	-0.072
M1[confraria]	1.000	(constrained)	.			
_cons	-3.816	3.135	-1.22	0.224	-9.961	2.329
**Choice: Exit**						
Bonding communication	0.332	0.659	0.50	0.615	-0.960	1.623
Bridging communication	2.284	0.999	2.29	0.022	0.326	4.242
Linking communication	-0.171	0.209	-0.82	0.414	-0.579	0.238
Communication informal leaders	-0.871	0.591	-1.48	0.140	-2.029	0.286
Bonding trust	-1.407	0.982	-1.43	0.152	-3.331	0.517
Bridging trust	-0.687	0.995	-0.69	0.490	-2.638	1.264
Linking trust	0.336	0.215	1.56	0.119	-0.086	0.757
Dependence on marine resources	-0.066	6.496	-0.01	0.992	-12.798	12.666

Gender	1.503	0.953	1.58	0.115	-0.365	3.372
Fisher identity	-0.912	0.764	-1.19	0.233	-2.409	0.586
Social capital	0.355	0.341	1.04	0.297	-0.313	1.023
Inequality	-0.215	0.138	-1.56	0.118	-0.485	0.054
Risk attitude	-0.070	0.142	-0.49	0.623	-0.348	0.208
Participation in decision making	-0.249	0.429	-0.58	0.562	-1.090	0.592
Job attachment	-0.757	0.295	-2.57	0.010	-1.335	-0.179
SES knowledge	0.234	0.325	0.72	0.472	-0.403	0.870
Income	0.308	0.250	1.23	0.219	-0.183	0.799
Household size	0.485	0.424	1.14	0.252	-0.346	1.316
Fishing assets	0.262	0.393	0.66	0.506	-0.510	1.033
Age	0.008	0.022	0.35	0.723	-0.036	0.052
Livelihood diversity	0.198	0.549	0.36	0.719	-0.878	1.273
Training activities	1.379	0.654	2.11	0.035	0.097	2.662
Fishing experience	-0.050	0.023	-2.15	0.032	-0.096	-0.004
M1[confraria]	1.000	(constrained)	.			
_cons	3.700	2.614	1.42	0.157	-1.423	8.823
var (M1[confraria])	1.602	0.877			0.548	4.685

Measures of fit

Log likelihood	-305.721
Log pseudolikelihood	-304.598

SI References

1. Pita, P., Fernández-Márquez, D., Antelo, M., Macho, G. & Villasante, S. Socioecological changes in data-poor S-fisheries: A hidden shellfisheries crisis in Galicia (NW Spain). *Mar. Policy* **101**, 208–224 (2019).
2. García-Lorenzo, I., Cabaleiro-Casal, M. J. & Varela-Lafuente, M. M. Fishermen's associations of the small-scale fisheries: Study applied to the participation in the Cofradías of Galicia (NW Spain). *Ocean Coast. Manag.* **178**, 11 (2019).
3. Macho, G., Naya, I., Freire, J., Villasante, S. & Molares, J. The key role of the barefoot fisheries advisors in the Co-managed TURF system of galicia (NW Spain). *Ambio* **42**, 1057–1069 (2013).
4. Villasante, S. *et al.* The Implementation of the Landing Obligation in Small-Scale Fisheries of Southern European Union Countries. in *The European Landing Obligation: Reducing discards in complex, multi-species and multi-jurisdictional fisheries* (eds. Uhlmann, S. S., Ulrich, C. & Kennelly, S. J.) 438 (2019).
5. Cinner, J. E. & Barnes, M. L. Social Dimensions of Resilience in Social-Ecological Systems. *One Earth* **1**, 51–56 (2019).
6. Cinner, J. E. *et al.* Building adaptive capacity to climate change in tropical coastal communities. *Nat. Clim. Chang.* **8**, 117–123 (2018).
7. Barnes, M. *et al.* The Social Structural Foundations of Adaptation and Transformation in Social-Ecological Systems. *Ecol. Soc.* **22**, 14 (2017).
8. Barnes, M. L. *et al.* Social determinants of adaptive and transformative responses to climate change. *Nat. Clim. Chang.* (2020). doi:10.1038/s41558-020-0871-4
9. D'agata, S. *et al.* Multiscale determinants of social adaptive capacity in small-scale fishing communities. *Environ. Sci. Policy* **108**, 56–66 (2020).
10. Cinner, J. Coral reef livelihoods. *Environ. Sustain.* **7**, 65–71 (2013).
11. Cinner, J. E. . *et al.* Changes in adaptive capacity of Kenyan fishing communities. *Nat. Clim. Chang.* **5**, 872–876 (2015).
12. Daw, T. M. *et al.* To Fish or Not to Fish: Factors at Multiple Scales Affecting Artisanal Fishers' Readiness to Exit a Declining Fishery. *PLoS One* **7**, 10 (2012).
13. Cinner, J. E., Daw, T. & Clanahan, T. R. M. C. Socioeconomic Factors that Affect Artisanal Fishers ' Readiness to Exit a Declining Fishery. *Conserv. Biol.* **23**, 124–130 (2008).
14. Muallil, R. N. *et al.* Willingness to exit the artisanal fishery as a response to scenarios of declining catch or increasing monetary incentives. *Fish. Res.* **111**, 74–81 (2011).
15. Mortreux, C. & Barnett, J. Adaptive capacity: exploring the research frontier. *Wiley Interdiscip. Rev. Clim. Chang.* **8**, 1–12 (2017).
